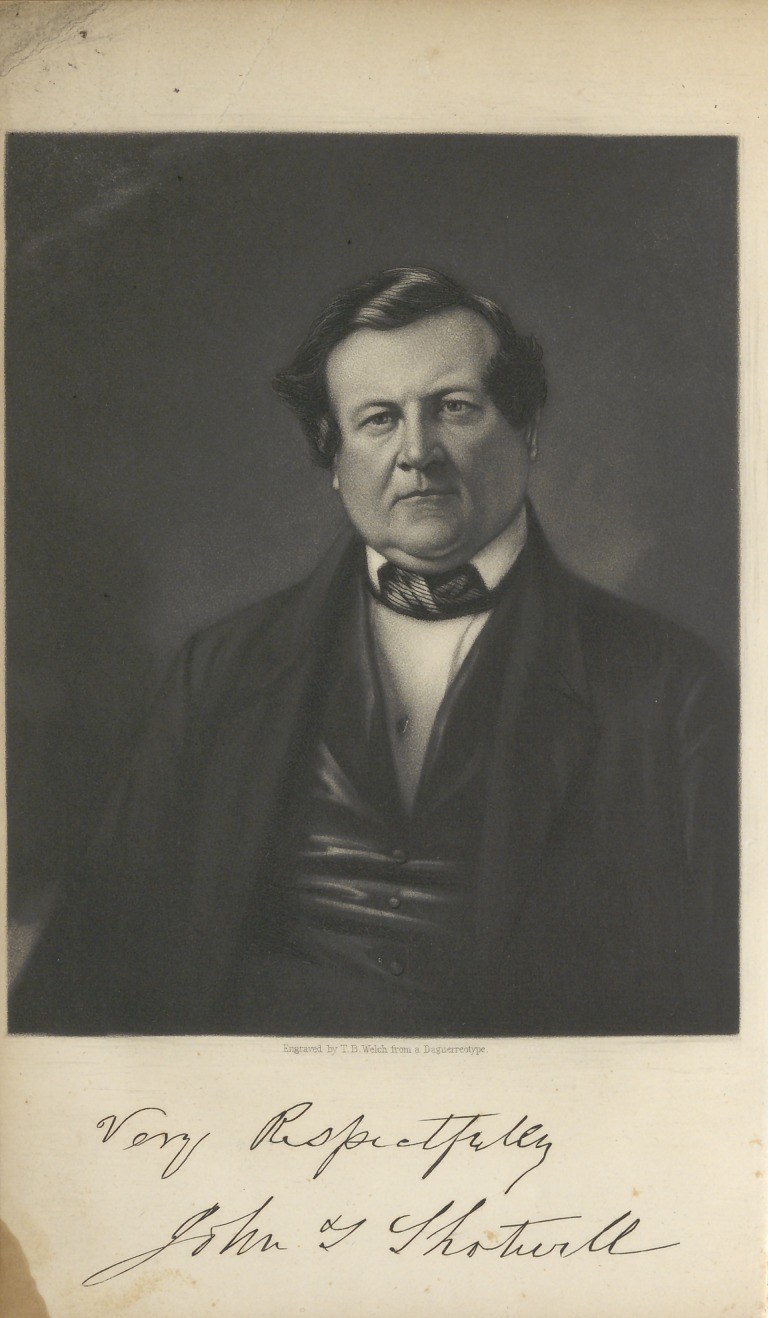# Biographical Sketch of Prof. Shotwell

**Published:** 1857-09

**Authors:** 


### BIOGRAPHICAL SKETCH OF PROF. J. T. SHOTWELL.

The medical profession offers to its members, when faithful to their duties, not only extraordinary means and opportunities of obtaining the respect and esteem of the cultivators of science, but also of acquiring the warm atttachment and affectionate gratitude of a circle of friends, more or less extensive, in proportion to the extent of their practice. This, although one of the most desirable blessings of life, is, to the physician, frequently the cause of its premature termination. Of these remarks, the subject of this sketch furnishes a very significant example.

John T. Shotwell was born in Mason County, Kentucky, on the 10th of January, 1807. His parents were immigrants from New Jersey, at an early period in the history of the West, and were among those who established its character as the home of hardy, brave, enterprising men of indomitable perseverance; and of kind-hearted, true-hearted, womanly women.

His father was a farmer during the early period of the life of his three sons; and his example, as well as his precepts, laid a foundation in their characters for the industrious habits in their several professions, by which they were distinguished.

A love of literature in early life, was remarked in the character of John, the eldest son, and his father determined to give him the best opportunities which the West afforded for the cultivation of this taste. This, with other reasons, induced the removal of the family to Lexington, where he entered the Transylvania University in 1822 and graduated in 1825, with so high a reputation for scholarship, that the late Dr. Drake, with that appreciation of character by which he was distinguished, was desirous to have him embrace the medical profession, and offered all the aids and advantages within his control, to qualify him for that excellence which he foresaw awaited him in that profession.

He entered the office of Dr. Drake in 1826, and continued his pupil until 1830, when he became his partner. His health having been impaired during his pupilage by too incessant devotion to his studies, he was induced in the spring of 1829 to try the effects of a journey in a more southern climate for its restoration.

He went first to New Orleans, and from thence journeyed on horse-back through several of the Southern States and the Indian Nation. This journey, although attended with much fatigue and exposure, greatly improved his health ; and his habit of observation, and of making the result of his observations practically useful in the principal pursuits of his life, added strength to his mind in proportion as exercise in open air added to the strength of his body. He became a graduate of Medicine at the Medical College of Ohio in 1832, and having dissolved his connection with Dr. Drake, he commenced practice with the advantages derived from that connexion and the reputation he had already acquired; and when the cholera visited this city, in that year, he became most extensively known as a successful practitioner, and as characterized by that amenity of disposition and anxiety for success in his treatment of cases, which convert patients into friends, and inspire that confidence which facilitates the labors of the physician, as well through a strict observance of his directions, as from the curative influence of faith and hope.

He married in 1832, Miss Mary Ward Foote, daughter of John P. Foote, Esq., of this city, and during the remainder of his life, displayed those amiable qualities as a husband which won so strongly the attachment of all who knew him, both as a physician and a man.

In the year 1832 Dr. Shotwell was appointed adjunct Professor of Anatomy to his friend, the celebrated Dr. Cobb who then occupied the chair of Anatomy in the Medical College of Ohio, and whose fame as a teacher of that science was rapidly extended throughout the United States, and excited competition in other institutions to obtain the advantages of his talents. Among the offers of chairs made him in other cities, he chose to accept that of the Medical Institute at Louisville, Ky., in which he remained until his bodily health became so affected by his labors as to necessitate an abandonment of the duties of a medical instructor. His resignation of the professorship he held in the Medical College of Ohio was followed by that of all the other professors except Dr. Locke, who was in Europe at that time, and Dr. Shotwell who was appointed to succeed Dr. Cobb.

He commenced his lectures on Anatomy at the session of 1837–8, and except for one session, continued during the remainder of his life, among the most highly esteemed members of the Faculty; increasing annually in reputation, and in the power of aiding the attractions of the institution which he adorned.

The strong inclination to devote his practice exclusively to surgery, which would naturally be developed by devotion to the duties of his chair, induced him, in 1842, to visit Europe for the purpose of learning from the celebrated surgeons of France and England, any improvement which their extensive practice had enabled them to make in their profession, a knowledge of which would be useful to him as an operator. He considered that his journey had been successful, and that the advantages thus obtained were a full compensation for the loss of time and expense of the journey.

For the purpose of relinquishing all except surgical practice, he formed a partnership with his former pupil Dr. H. E. Foote, intended to be relieved, by this association, from as much of his ordinary regular practice as possible. Had he been permitted to carry this plan into *full* effect, his valuable life might perhaps have been spared. But during the cholera season of 1850, his regular patients would not dispense with his attendance, and many others, in their anxiety to obtain his services, added to the fatigue, labor, and exposure he was compelled to undergo. His own health soon yielded to his solicitude for the health of others, and one evening, on his return home from a very arduous series of labors, he found himself unable to do more, and went to bed with a determination to take that rest which he found had become absolutely essential to the preservation of his power to continue his usefulness. Foreseeing that calls would be made upon him during the night, his family determined that all of them should be dismissed without his knowledge. This was done with several applicants, but at length one of them insisted so strongly on seeing him, and asking at his bedside for advice in the case of his wife whom he considered in a peculiarly dangerous condition, that he was permitted to do so, and being there, he represented to the doctor that the life of his wife depended on seeing him—that her confidence in his skill was such that the mere sight of him would be more efficacious for her recovery than any medicine that could be administered. The doctor could not resist this appeal, and left his bed, to which he returned after his visit, but never left again. In the eloquent memoir of him, by his attached and steady friend Dr. J. L. Vattier, the following statement is given of the commencement and termination of his disease:

"On the morning of the 14th of July he visited the Hospital.

On his way home, finding himself quite unwell, he called at my house to rest himself. He looked pale and haggard : remained twenty minutes and left for his home. That night he was summoned to attend a patient on Third street, and while preparing for the visit, was assailed with unmistakable symptoms of cholera. He, however, attended the call, and while yet at the house of his patient, the symptoms becoming more aggravated in their character, he was forced to resort to medical remedies to enable him again to reach his home.

The next morning his friend, that excellent physician, Dr. Walcot Richards, was summoned to attend him, and continued to have the principal charge of his case up to the time of his death. I saw him frequently, as did many others of his numerous friends.

His disease assumed a malignant character, and he was for a while considered in imminent danger ; at last, however, medical skill obtained the mastery, and he commenced to convalesce, and continued to do so for several days. He was patient under suffering, and looked forward with hope to a speedy restoration to health , but this hope proved false, for with him all life's scenes were fast drawing to a termination. The improvement in his condition was not destined to be permanent ; reaction became too violent : congestion of the brain set in, and though every means was resorted to that medical skill could suggest, death closed the scene at 11½ o'clock, P. M., on the 23d of July."

His death was regarded as a public calamity by all classes of citizens ; and a stranger, on seeing the immense assemblage at his funeral, anxious to pay the last tribute of affection and respect, would have understood that a great man had fallen, and if he had inquired what manner of man, and who it was that had such power after death over the hearts and feelings of such a multitude, he would have been told that a good man was lost to a numerous body of friends, who justly appreciated his virtues and talents ; that a heavy affliction was laid on the poor who had experienced their benefits, and that the science of medicine had lost one of its most promising cultivators, in the death of John T. Shotwell.

From among the numerous Obituary Notices called forth on the occasion, we select the following as expressions of public feeling, and testimonials of the power which a physician who fulfills the duties of his profession with diligence, and in a Christian spirit, will obtain over the affections of all who know him ; and of the deep regret which attends the loss of one so useful and so free from all suspicion of any taint of selfishness in his character.

Death of Dr. John T. Shotwell.—Our city was thrown into general gloom yesterday upon the announcement of the death of the eminent man whose name heads this article, and expressions of sorrow fell from every lip when the mournful tale was told. The rich and the poor, the high and the low, had the tribute of a tear, and coming from hearts warmed by kindly recollections, these tears told a more expressive tale of deeply implanted grief, than could the plumed pomp and sable pageant that attends upon the demise of the loftiest in the land. For him sprung the sorrow of the soul, and the fountains of heart-born grief gave out their tribute to pure worth, while charity stood beside the breathless corse and wept for the like she will never look upon again.

It is needless to dwell upon his virtues, for they are written upon the hearts of all who knew him, and he made the indelible stamp of a man upon the minds of all with whom he associated. Endowed with an extraordinary degree of strong common sense, and having an intuitive knowledge of human nature, he endeared himself to all, whether socially or professionally, and a moment's acquaintance was the foundation of a life-time love. The poor hovel of poverty shared his kindly attention alike with the glittering palace of affluence—and hundreds will weep as the solemn funeral train passes to-day, even as the loving child bends under the affliction of a father's loss. Like the Angel of Mercy, it needed but the voice of pain to arouse that good old heart of his,—a heart whose sympathetic responses were not awakened by the sordid prospects of worldly gain. He was a man of the world in one sense, but in another sense, a man whose endearing attributes elevated him above the wiles and the ways of the world. He was all that God may make of man, and may He to whom humanity bends the knee for mercy, hear the prayers that ascend from many a heart to-day.—*Commercial.*

Dr. Shotwell, a well-known and respectable physician of this city, died yesterday morning. He was a man in high esteem, in this community, of rare medical attainments, and enjoyed an extensive practice in the line of his profession. His loss will bring sorrow to hundreds of hearts, and leave a vacancy in the ranks of the medical profession, which years will not suffice to refill.—*Atlas.*

Dr. John T. Shotwell.—The announcement of the death of this universally popular physician and man, under the Obituary head in another column, will be read with emotions of the deepest regret by those who have not already been grief-stricken with the sad intelligence through other channels.

The death of Dr. Shotwell has cast a deep gloom over the whole community, and well it might, for perhaps no man was more generally known or endeared to a larger circle of acquaintances. His innate goodness of heart, his social qualities and winning manners had rendered him universally popular. There was an atmosphere of good nature, warm and generous impulses always surrounding him, which made him the favorite of every circle. His presence and kind manner always brought a cheering ray of sunshine into the sick-room—and the despondent patient caught an inspiration from his presence—a cheering hope that was worth more than a score of ordinary prescriptions.

Dr. Shotwell has been known long and well as a skillful and highly successful practitioner of medicine,—and a popular teacher of his favorite science. During the long period in which he filled the chair of Professor as one of the faculty of the Ohio Medical College, he was universally popular with all classes of students, and was as general a favorite in the lecture room, as in other walks of life.

His untimely death, in the fulness of the noon of life, has created a void which can not be filled, and those to whom he was endeared will not soon forget the many good qualities which made him cherished by a host of sorrowing friends.—*Dispatch.*

---

[During the sessions of 1848–49, and 1849–50, Prof. Shotwell occupied the chair of "Anatomy and Physiology," in the "Ohio College of Dental Surgery," and, by his urbanity of manner, and his zeal and energy as a teacher, he made a favorable impression on the hearts of his pupils and fellow professors, which can never be effaced.

> "Time but the impression deeper makes
> As streams their channels deeper wear."

His heart was so fully impressed with the importance of the science which he taught, as lying at the foundation of all medical knowledge, that, whether his class was large or small, he manifested the same untiring energy. Having attended his lectures in the crowded classes of the "Medical College of Ohio," and again in the smaller classes incident to dental schools, we can bear ample testimony on this point. His pupils, whether medical or dental, will not soon forget his teachings.]—*Eds. Reg.*

## Figures and Tables

**Figure f1:**